# Which types of social support matter for Black sexual minority men coping with internalized homophobia? Findings from a mediation analysis

**DOI:** 10.3389/fpsyg.2024.1235920

**Published:** 2024-02-06

**Authors:** Hayden C. Dawes, Tiffany M. Eden, William J. Hall, Ankur Srivastava, Denise Yookong Williams, Derrick D. Matthews

**Affiliations:** ^1^School of Social Work, University of North Carolina at Chapel Hill, Chapel Hill, NC, United States; ^2^Department of Public Mental Health, Bloomberg School of Public Health, John Hopkins University, Baltimore, MD, United States; ^3^Global Public Health, University of North Carolina at Chapel Hill, Chapel Hill, NC, United States

**Keywords:** internalized homophobia, depression, Black sexual minority, social support, mediation

## Abstract

**Background:**

Minority stress theory views social support as a protective factor against the effects of minority-specific stressors like internalized homophobia (IH) on mental health in sexual minority populations. However, much of the empirical validation of this theory has been conducted within predominantly White samples, resulting in a limited understanding of how the theory applies to Black sexual minority individuals. Current examinations of social support fail to capture the nuances of how Black sexual minority men may access support systems differently, resulting in a need to investigate how social support, IH, and mental health operate for Black sexual minority men. This study examined relationships between IH, depression, and different types of social support (i.e., family, friends, Black community, gay community) using a mediation model.

**Methods:**

We used data from the POWER (Promoting Our Worth Equity and Resilience) Study, which recruited Black sexual minority men at Black Pride events across six cities in the United States from 2014 to 2017, to test four mediation pathways concurrently in Stata 17. Participants (*N* = 4,430) completed a questionnaire assessing a variety of health and life domains, including depression symptoms, internalized homophobia, and social support.

**Results:**

IH was positively associated with depression. Lower levels of family, friend, and Black community support were all positively associated with depression symptoms. Additionally, IH was positively associated with all types of support. Finally, family, friend, and Black community support partially mediated the relationship between IH and depression.

**Conclusions and implications:**

Results suggest that the relationship between social support and depression is complex for Black sexual minority men. Findings suggest family support is an important factor for clinical intervention efforts targeting depression, and that gay community support systems should assess how their environments can better support Black sexual minority men. Overall, findings demonstrate the necessity of future examination of how social support functions differently within Black sexual minority communities.

## Introduction

In the United States, up to 9.4% of adults (National Center for Health Statistics, 2019; Jones, 2021), identify with a sexual minority identity (e.g., gay, bisexual, pansexual, queer) or report same-sex behavior. Of these sexual minorities, approximately 13.4% are Black or African American (United States Census Bureau, 2022). Despite comprising a small percentage of the U.S. population, Black sexual minority men (BSMM) face pronounced health disparities, including high rates of depression (Vincent et al., 2020) compared to their White peers. Leading explanations for high rates of behavioral health disparities facing BSMM point to the intersectional compounding of stressors (e.g., discrimination, violence, exclusion, internalized stigma) stemming from overlapping systems of oppression: racism and heterosexism (Graham et al., 2011, 2016; English et al., 2021). Growing evidence has elucidated a predictive relationship between intersectional, oppression-based stressors and negative behavioral health outcomes for BSMM, including depression and suicidality (Lytle et al., 2014; Sutter and Perrin, 2016). These threats have significant implications for the quality of life, social functioning, and the very lives of BSMM; however, there is a paucity of evidence regarding psychosocial mediators and moderators that could inform the development of interventions to prevent or mitigate these health threats.

### Minority stress theory

Minority stress theory, originally presented by Brooks (1981) and then later advanced by Meyer (2003) been the prevailing framework applied to examine sexual minority stress. However, a key limitation of the data that supports the framework is its overwhelming dependency on predominantly White samples (Meyer, 2010; Moradi et al., 2010a,b). Key stress-related mechanisms and protective factors may operate differentially when it comes to Black populations. Minority stress theory posits that psychosocial support moderates the relationship between minority-specific stressors, like internalized homophobia, and mental health outcomes, such as depression. Black sexual minorities who experience internalized homophobia may access support systems differently than White sexual minorities, given specific racialized and cultural factors impacting sexual minority identity self-disclosure (Friedman et al., 2019).

### Internalized homophobia, depression, and social support

In recent decades, theory and evidence have shown that internalized homophobia is a prominent psychosocial stressor contributing to depression in sexual minority men (Shidlo, 1994; Rowen and Malcolm, 2003; Newcomb and Mustanksi, 2010; Heiden-Rootes et al., 2020). Internalized homophobia is a cognitive, emotional, and behavioral process in which sexual minority individuals adopt the negative beliefs and attitudes that permeate society about sexual minority people (Amola and Grimmett, 2015). Research suggests that individuals often seek support from those in their social networks (e.g., friends, family members, communities) when faced with stressors (Pearlin and Bierman, 2013). However, sexual minorities and BSMM, in particular, may have fewer or unhelpful sources of social support because they may be more socially isolated due to shame, identity concealment, or past experiences with discrimination, rejection, and/or microaggressions (Meyer, 2010; Arnold et al., 2014; Smallwood et al., 2017).

### Importance of mediation analysis for sexual minority populations

To examine the intermediate variables that influence the pathways between sexual minority-specific stressors and their outcomes, it is important to conduct mediation analyses. Mediation analyses are employed when an independent variable is posited to create change in an intervening variable (i.e., mediator) that, in turn, causes a change in an outcome (MacKinnon et al., 2002). A systematic review (Argyriou et al., 2021) identified 40 studies that examined mediators with sexual minority populations. Out of these 40 studies, only 9 of them studied social support as a mediator to depression, and none of them explored these mediators with samples of primarily BSMM.

A recent study (Puckett et al., 2015) of sexual minority adults found that social support mediated the relationship between internalized homophobia and mental health problems (i.e., depression, anxiety, and suicidal ideation), with higher internalized homophobia being associated with lower social support and lower social support being associated with more psychological distress. However, this study used a predominately White sample, and did not include an examination of multiple minorities. Additional research is needed to explicate these relationships, especially with BSMM, considering the multiple marginalizations and high rates of behavioral health disparities faced by this population (Vincent et al., 2020; English et al., 2021). Furthermore, much of the extant evidence relies on studies with largely White samples, with limited representation of BSMM (Meyer, 1995, 2003; Szymanski et al., 2008; Moradi et al., 2010a,b). Clarifying mechanisms underlying the relationships between internalized homophobia and mental health outcomes for BSMM is imperative, as these factors may operate differently for this unique group.

### Purpose of current study

Given the critical gaps in knowledge on key mediating factors in BSMM’s pathways of psychosocial stressors and depression disparities, the purpose of this study was to examine which types of social support may mediate the relationship between internalized homophobia and depression symptoms among BSMM. This study examined various types of social support as potential mediators, including social support from various relationship groups including family, friends, sthe gay community, and the Black community. Based on prior research (though very limited with BSMM), we hypothesized that (a) internalized homophobia would be positively related to depression, (b) internalized homophobia would be inversely related to social support, and (c) social support would be inversely related to depression symptoms.

## Methods

This study used data from the POWER (Promoting Our Worth, Equity, and Resilience) project (Eaton et al., 2018), a cross-sectional study of BSMM (*N* = 4,430) recruited at queer Black pride events in Atlanta, GA; Detroit, MI; Houston, TX; Memphis, TN; Philadelphia, PA; and Washington, DC from 2014–2017. At each pride event, the study team created an intercept zone, where individuals were approached, and invited to participate in the research study. Participants were provided $10 for survey completion.

To be eligible for the study, individuals must have been assigned male sex at birth, had a male sexual partner in their lifetime, self-identified as Black or African American, and be at least 18 years old. Survey assessments were anonymous. Eligible participants were provided informed consent. Participants completed an anonymous, computer-assisted self-interview (CASI) using an electronic tablet. Each participant was given a unique code. This code was based on a sequence of letters and numbers from the participant’s name, a family member’s name, birth date, and state of birth. Participants completed the self-report survey about demographics (racial/ethnic identity, age, income, education level, sexual identity), internalized homophobia, social support, and depression symptoms. Participants completing more than one survey were identified. Further detail regarding the process used to identify and remove repeated respondents has been described elsewhere (Eaton et al., 2018). The University of Pittsburgh Institutional Review Board approved all study procedures.

### Measures

*Socio-demographic characteristics* were collected. Participants were asked about their age, highest level of education, annual income, and sexual orientation. Sexual identity was measured with the item, “Which of the following do you identify with?” Options included: gay/same gender loving, heterosexual or straight, bisexual, or other. Education was assessed with the item, “What is the highest level of education you completed?” Annual income was asked, “What would you say is your annual personal income before taxes?” Age and income level were treated continuously. Education, sexual orientation, year, and city of data collection were treated as categorical variables.

*Internalized homophobia* was measured using the 9-item Internalized Homophobia Scale (IHS; Herek et al., 1997; e.g., “I feel alienated from myself because of being gay/bisexual”), with 5-point response options ranging from *Strongly Agree* (0) to *Strongly Disagree* (4). The internal consistency reliability was very good (*α* = 0.92). Item scores were averaged, and ranged from 0 to 5, with the higher values indicating a higher degree of internalized homophobia. IHS has been applied to BSMM in previous research (Dawson et al., 2019) and had a high internal reliability within that sample (*α* = 0.88).

*Social support* from four sources (Black community, family, friends, and the gay community) was measured by asking, “To what degree do you feel you receive support from….” Each source of support was a separate item. Response options included *None*, *A little*, *Somewhat*, and *A lot* (coded as 0 to 4) for each source of support. Support items were reverse-coded in the analysis, with higher numbers indicating lower social support.

*Depression symptoms* in the past week were measured using the 10-item version of the Center for Epidemiological Studies (CES-D-10; Andresen et al., 1994) with 4-point response options ranging from *Rarely or none of the time* to *Most or all of the time (0 to 3)*. Question scores were then summed to provide an overall score ranging from 0 to 30. A higher score indicates greater depression. The internal consistency reliability was acceptable (*α* = 0.75). The scale has been widely used and tested with Black and sexual minority populations (Conerly et al., 2002; L. F. Graham et al., 2011).

### Data analysis

Data preparation, management, and analysis were performed using Stata 17. Descriptive statistics were used to describe all variables and the sample. To examine the relations between the primary independent variable (internalized homophobia), mediating variables, and outcome of depression, multiple pathways were analyzed, including a series of regressions, following the framework outlined by Baron and Kenny (1986). Before analysis, several assumptions were examined, and diagnostic tests were performed, including linearity between the variables, errors that are normally distributed, homoscedasticity of errors, and independence of the observations. Listwise deletion of missing data was performed with 4.6% (*n* = 236) of missing observations. Indirect effect analysis was completed using the RMediation package (Tofighi and MacKinnon, 2011). Total effects were also calculated.

## Results

### Sample description

Table 1 contains demographic information. The mean age of the participants was 31.1 years (*SD* = 10.1). In terms of sexual orientation identity, 79.7% (*n* = 4,089) of the sample identified as gay or same-gender loving, followed by bisexual (17.5%; *n* = 900), and another identity (1.5%; *n* = 77). Further, 36.2% (*n* = 1842) of participants reported having completed some college or technical school, followed by college graduate (35.7%; *n* = 1,815), High school/GED (21.0%; *n* = 1,068), and less than High school/GED (7.1%; *n* = 361). Participants also reported on average, 6.7 (*SD* = 4.9; range 0–30) depression symptoms, and on average, 1.3 (*SD* = 1.0; range 0–4) score on internalized homophobia.

**Table 1:** Sample characteristics.

Age, mean (SE)	31.1 (*SD* = 10.1)
Education, No. (%)	
<High school/GED	361 (7.09)
High school/GED	1,068 (21.00)
Some college or technical school	1,842 (36.22)
College graduate	1,815 (35.68)
Sexual orientation identity, No. (%)	
Gay or same-gender loving	4,089 (79.66)
Bisexual	900 (17.53)
Other (e.g., heterosexual, MSM)	77 (1.50)
Annual income	
$0–9,999	1,023 (20.24)
$10,000 – $29,999	1,344 (26.59)
$30,000 – $49,999	1,332 (26.36)
$50,000 – $69,999	385 (14.54)
$70,000 – $89,999	735 (7.62)
$90,000 – and up	235 (4.65)
Depression symptoms^a^, mean (SD)	6.66 (4.89)
Internalized homophobia^b^, mean (SD)	1.34 (1.01)
Types of Support^c^, mean (SD)	
Lower Friend	0.71 (1.05)
Lower Gay Community	1.05 (1.11)
Lower Black Community	1.25 (1.10)
Lower Family	1.03 (1.13)
City	
Atlanta	1,472 (28.67)
Detroit	658 (12.82)
Houston	1,135 (22.11)
Memphis	84 (1.64)
Philadelphia	709 (13.81)
Washington, DC	1,076 (20.96)
Year of data collection, No. (%)	
2014	1,500 (29.22)
2015	1,725 (33.60)
2016	1,331 (25.93)
2017	578 (11.26)

Analytic sample *n* = 4,430; ^a^Range = 0 to 30, with higher values indicative of greater depression symptoms. ^b^Range = 0 to 4, with higher values indicative of greater internalized homophobia. ^c^Range = 0 to 3, with higher values indicative of lower levels of support.

### Internalized homophobia, social support, and depression symptoms

**Figure 1:**
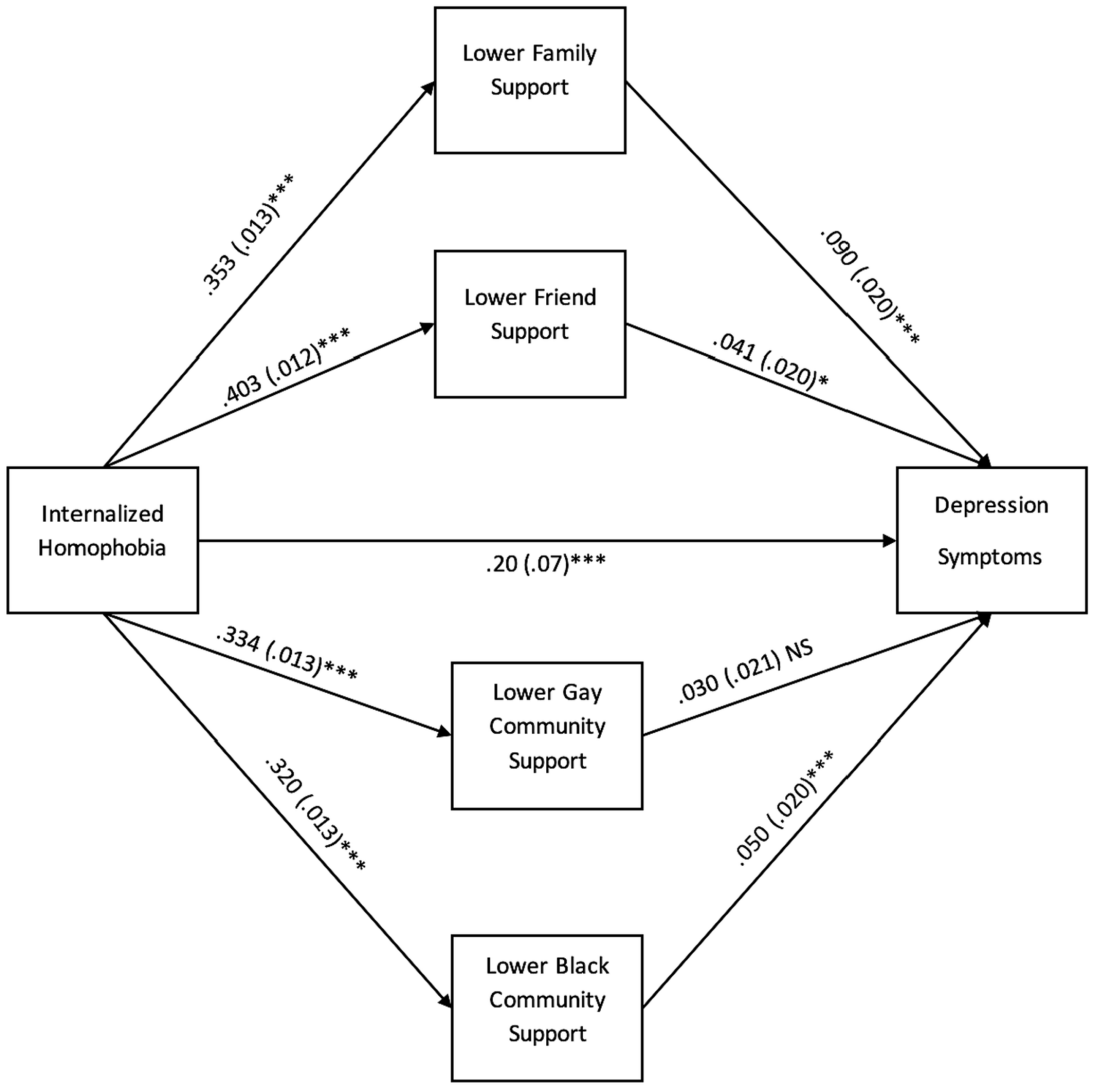
Mediation pathways to depression symptoms through multiple types of support. *p < 0.05, **p < 0.01, *** *p* < 0.001, NS, non-significant. Standard errors (*SE*) are in parentheses. All coefficients are standardized.

We conducted a multiple mediation model to examine types of social support mediating the relationship between internalized homophobia and depression symptoms. Intercorrelations between variables in the mediation models are presented in Table 2. As shown in Figure 1, the direct effect of internalized homophobia was positively associated with depression symptoms. Lower family, friend, and Black community support (independent of each other) were associated with depression. The pathways to depression, indirect effects, and total effects of these mediators are shown in Table 3 symptoms.

**Table 2:** Correlations between primary study variables.

	1	2	3	4	5	6
1. Internalized homophobia	1.0					
2. Depression symptoms	0.29	1.0				
3. Lower friend support	0.44	0.25	1.0			
4. Lower gay community support	0.36	0.20	0.58	1.0		
5. Lower Black community support	0.32	0.19	0.51	0.72	1.0	
6. Lower Family support	0.37	0.23	0.67	0.50	0.51	1.0

All values were significant at the 0.001 alpha level.

**Table 3:** Pathway analysis to depression.

	*TE*	*β*	*SE*	95% CI
Lower family (LFM)	0.232			
IH → LFM		0.353***	0.013	[0.328, 0.378]
LFM → DS		0.090***	0.019	[0.053, 0.127]
IE		0.032	0.007	[0.019, 0.045]
Lower friend (LFD)	0.217			
IH → LFD		0.403***	0.012	[0.380, 0.426]
LFD → IH		0.040*	0.020	[0.002, 0.080]
IE		0.017	0.008	[0.001, 0.032]
Lower gay community (LGC)	*NS*			
IH → LGC		0.334***	0.013	[0.309, 0.360]
LGC → DS		0.030 (*NS*)	0.021	[−0.012, 0.069]
IE		0.009 (*NS*)	0.007	[−0.004, 0.023]
Lower Black community (LBC)	0.214			
IH → LBC		0.320	0.013	[0.291, 0.343]
LBC → DS		0.050***	0.020	[0.006, 0.085]
IE		0.015	0.006	[0.002, 0.027]

*R*^2^ = 13.6% of the variance. Values are standardized path coefficients. IE, Indirect Effect; TE, Total Effect for significant variables; CI, Confidence Interval; DS, Depression Symptoms. *p < 0.05, **p < 0.01, ***p < 0.001, NS, non-significant.

### Control variable results

We adjusted for age, sexual orientation, education level, income level, city of data collection, and year of data collection to the outcome of depression. These individual predictors were examined and indicated the following coefficients and *p* values: age (*β* = 0.031, *SE* = 0.01, *z* = 2.28, *p* = 0.022), sexual orientation (*β* = −0.02, *SE* = 0.01, *z* = −2.12, *p* = 0.034), education (*β* = −0.059, *SE* = 0.02, *z* = −3.89, *p* = 0.00), income level (*β* = −0.13, *SE* = 0.014, *z* = −8.66, *p* = 0.00), city of data collection (*β* = −0.02, *SE* = 0.014, *z* = −1.48, *p* = 0.14), year of data collection (*β* = −0.002, *SE* = 0.014, *z* = 0.20, *p* = 0.84).

## Discussion

This is the first study to our knowledge to examine the relationship between internalized homophobia and depression as mediated by various types of support in a large sample of BSMM. Overall, the study extends our knowledge of how varying types of support may operate with these men in the context of internalized homophobia and depression. These findings are consistent with our hypotheses that family, friend, and Black support partially mediate the relationship between internalized homophobia and depression symptoms. However, we did not find evidence that lower gay community support is independently associated with depression symptoms or mediates the relationship between internalized homophobia and depression symptoms.

The mainstream gay community’s cultural norms within the United States largely privilege White Eurocentric norms (Han, 2007). Thus, it is unsurprising that there was no significant effect for gay community support relating to depression for Black sexual minority men. In contrast, the other forms of support were significant. Within the United States, the gay community, including gay advocacy organizations (Adams, 2019) and online dating platforms (Callander et al., 2015; Wade et al., 2021), have long been criticized for ascribing to mostly White standards (e.g., music, art) and social values (e.g., beauty esthetics, hyper-secularism) that do not serve Black men along with other racial minorities (Han et al., 2017; Pingel and Bauermeister, 2018; Worthen, 2018). Therefore, these environments, such as dating apps, social clubs, and informal networks, largely remain alienating to the culturally-specific psychosocial needs of queer men of color. For this current sociocultural landscape to shift, gay community events, platforms, and civic and social groups must make additional efforts to become more racially inclusive, affirming by further integrating the cultural mores of BSMMn and having leadership positions for BSMM. These issues are often found in societies with histories of colonization, enslavement, and/or modern forms of social oppression stemming from White European supremacy (e.g., Logie and Rwigema, 2014; Durban, 2021; Wahab, 2021).

Family support had the strongest association among all the mediators, demonstrating that familial relationships are a salient factor for mitigating depression in this population. Yet, examining and leveraging family ties to improve BSMM mental health is underutilized (Dawes et al., 2022). Studies (Ryan et al., 2010; Swendener and Woodell, 2017; Boyd et al., 2021) have shown that family acceptance of sexual minorities, which includes identity affirmation, improved communication, and family activities, can have a robust effect on improving sexual minorities’ self-concepts (e.g., self-esteem), and longer-term mental health outcomes. Mental health service providers and systems should assess family factors and develop interventions centered around families, including the family of origin and extended families, among BSMM struggling with internalized homophobia and depression. The aims of these interventions should focus on improving weak family links and maintaining strong ones between BSMM and their families. With anti-sexual minority bias and stigma potentially operating as primary influences on deleterious relationships, they must be targeted. Therefore, interventions should recognize the harmful influence of homophobia and heterosexism by addressing discrimination and other anti-queer bias that BSMM and their families often internalize. Research (Pingel and Bauermeister, 2018) shows that Black people are largely a religious population group; therefore, affirming religious communities and leaders offer a critical platform for mental health practitioners and systems to create collaborative relationships. Church and other religious groups may be able to educate Black families in intervening to reduce BSMM’s depression and its adverse consequences.

### Implications for mental health systems and policy

Due to stigmatization and access to care issues, BSMM face inadequate mental health systems to meet their needs. Several barriers exist for BSMM to receive ideal treatment, such as payer source issues and unresponsive mental health systems and providers (Dawes et al., 2022). Low-income BSMM in particular may have limited access to mental health care due to relatively high-cost private pay session rates and overall disinvestment of community mental health centers (Morrissey and Goldman, 2020). Such barriers make it difficult to attend weekly or bi-weekly therapy sessions necessary for adequate depression treatment. Due to higher costs, residential or intensive outpatient groups may be an even greater challenge for those needing higher levels of treatment. Moreover, many mental health systems and providers choose not to take public or private insurance; conversely, when providers take insurance, BSMM often continue not to have access (Dawes et al., 2022). Thirty-four percent of the general public with private insurance report having difficulty locating an in-network therapist (Petersen, 2021). Furthermore, many BSMM work as contract employees, freelancers, or gig workers, who are not entitled to employer-sponsored health insurance or meeting income thresholds for public programs such as Medicaid (Gelles-Watnick and Anderson, 2021). Furthermore, public mental health funds may not provide the long-term treatment needed to address depression fully. These payer source issues create a sizeable obstacle regarding payer source targeting BSMM’s depression.

Mental health services and systems are largely led and provided by White heterosexual practitioners, creating a treatment milieu with few BSMM providers informing care (Callahan et al., 2018). These contemporary systems have been characterized as having policies and interpersonal biases that thwart affirming care for BSMM. To resolve this situation, recommendations include developing intersectional informed mental health training for providers and systems, supporting efforts to diversify the mental health workforce along the lines of sexual orientation and race, and developing systems policies that center BSMM’s needs and treatment concerns. Additionally, there are a growing number of individual and group-based mental health interventions focusing on intersectional oppression among BSMM that will need mental health system adoption for adequate further treatment proliferation (Jackson et al., 2022). Policymakers on the federal, state, and systems levels have a role in eliminating barriers to care, creating comprehensive mental health coverage policies, and ensuring that the mental health workforce is diverse and treatment approaches are intersectionally responsive to BSMM.

### Limitations

Although the study has several strengths, it has certain limitations regarding the generalizability and causal inference of study results. The sample was a convenience sample of BSMM who attended pride events; therefore, there is a possible selection bias in that BSMM who may have high rates of internalized homophobia would likely not attend a pride event. These findings are limited by the cross-sectional design, with the mediators, predictors, and outcomes being measured concurrently. Therefore, there are concerns regarding temporal ordering and causal inference. Finally, the measurement of support variables could be more robust by adding additional items to measure the multidimensionality of social support for BSMM more precisely.

### Future research

Future work should concentrate on longitudinal design and analysis to examine the temporal mechanisms of multiple forms of social support in mitigating internalized oppression and depression within BSMM. Further investigation is needed to identify the other formal social supports unique to queer Black communities that have not been identified or explored thus far, including Black spiritual community support, support from chosen family, or support from the queer Black community. Indeed, an intersectionality approach is needed to identify social stressors and oppressive systems affecting BSMM, investigate unique forms of internalized oppression, and develop effective interventions for these psychosocial stressors and mental health issues among BSMM. Strengthening this work with better detection of intermediate variables and their impact on depression prevention and clinical depression interventions for BSMM will promote mental health equity.

## Data availability statement

The raw data supporting the conclusions of this article will be made available by the authors, without undue reservation.

## Ethics statement

Ethical review and approval was not required for the study on human participants in accordance with the local legislation and institutional requirements. Written informed consent from the patients/participants or patients/participants’ legal guardian/next of kin was not required to participate in this study in accordance with the national legislation and the institutional requirements.

## Author contributions

HD conceptualized the study and led the study design. HD, TE, WH, AS, DW, and DM analyzed the data, interpreted the results, wrote, and edited parts of the manuscript. All authors approved the manuscript as submitted.
